# Human muscle and spinal activation in response to body weight loading

**DOI:** 10.1111/joa.13821

**Published:** 2023-01-31

**Authors:** Benjamin Clarke, Jannah Khalid Al‐Hammdany, Irene Di Giulio

**Affiliations:** ^1^ School of Basic and Medical Biosciences, Faculty of Life Science and Medicine King's College London London UK

**Keywords:** human standing, muscle activation, spinal activation, weight bearing

## Abstract

Human standing is the anatomical and functional framework for independent movement. The study of weight bearing during standing and movement is crucial to support the development of technology aimed at restoring independent gait, where unsupported weight bearing is still elusive. This study aims to determine muscle and spinal activation at different gravitational loads in young, healthy individuals to provide potential patterns of spinal stimulation for standing. Muscle activity was recorded with surface electromyography (EMG) from 18 healthy participants at different body angles while on a motorised plinth. The body angles tested and the relative gravitational loadings were: 0 deg (supine) corresponding to ~0% of total body weight (BW), 15 deg (~26% BW), 30 deg (~50% BW), 45 deg (~71% BW), 60 deg (~87% BW), 75 deg (~97% BW), upright on and off the plinth (~100% BW). The muscles recorded were soleus, gastrocnemius medialis and lateralis, tibialis anterior, adductor longus, peroneus longus, vastus medialis and lateralis, rectus femoris, sartorius, extensor digitorum longus, semimembranosus, semitendinosus, biceps femoris, gracilis, rectus abdominis, external oblique, erector spinae and latissimus dorsi. From the recorded muscle activity, spinal activation maps were calculated. Despite high variability in EMG data, the group muscle activity changed with body angle. Vastus lateralis became activated at 60 deg (~87% BW), soleus became activated at 75 deg, and the gastrocnemii at 90 deg. The spinal segments that showed significant differences in mean activation between angles were the fifth lumbar L5 and the first sacral S1 segments. The data from this study suggest that weight‐bearing independent standing could be achieved with increased activation of a limited number of superficial muscles tested, and 87% BW is a critical loading for increased muscle activation compared to the supine position. The spinal activation in the lower lumbar and sacral segments shows the involvement of these regions in maintaining weight‐bearing standing. By reproducing this pattern of muscle and spinal segment activity through tonic stimulation, we speculate that restoration of independent standing and walking may be possible for patients following spinal cord injuries.

## Graphical abstract

Muscle activity was recorded with surface electromyography to determine spinal activation at different gravitational loads. The fifth lumbar and first sacral segments were involved in maintaining weight‐bearing standing.

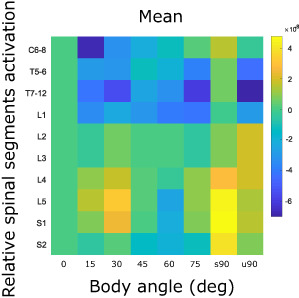

## 1 INTRODUCTION

Human standing is an important and complex task. It serves as the mechanical and control basis for other motor functions and requires regulation of weight bearing, balance and whole‐body control. Walking, for which standing is a prerequisite, shares these requirements and the two functions are closely interlinked. Balance is critical during initiation of gait, since the body mass is moved forward from a stable position so that the body can advance (Hall et al., 2010; Mancini et al., 2009), and during the single limb supported swing phase, where the centre of mass moves outside the base of support (Lugade et al., 2011). In healthy adults, control of standing and walking is regulated by the central nervous system at multiple levels. This includes supraspinal control, where descending motor tracts allow for voluntary movement and adjustments for changes in direction, speed and terrain (Drew et al., 2004; Rossignol et al., 2009); spinal circuits, which produce rhythmic stepping patterns (Barbeau & Rossignol, 1987; Frigon, 2012; Minassian et al., 2016; Rossignol et al., 2009); and peripheral sensory‐motor integration, which allows for appropriate modulation of spinal output to adapt stepping to the immediate environment (Edgerton et al., 2004).

Spinal cord injuries (SCI) can affect these mechanisms, leading to substantial loss of sensory and motor function, including loss of independent standing and walking. Novel treatments for SCI aim to restore this lost function, particularly independent gait, with spinal and nerve stimulation. Recent clinical and technological advancements have successfully reproduced rhythmic stepping patterns in individuals with SCI; however, independent weight bearing remains elusive. For example, ground‐breaking work from Harkema and colleagues showed that an individual with motor complete SCI could produce locomotor patterns when stimulated epidurally at the fifth lumbar (L5) and first sacral (S1) spinal segments (Harkema et al., 2011). In the same study, standing was initially achieved with 65% bodyweight support and was sustained during progressive reduction in support down to 5% (Harkema et al., 2011). After extensive training, the patient could achieve and maintain standing with mechanical stabilisation of the pelvis and knees and while holding on to a support frame. This evidence suggests that achieving independent function is within reach for patients with SCI. More recent inspiring work has shown that a more widespread stimulation of the spinal cord, including the lower thoracic segments, can achieve enhanced results. Weight bearing was achieved within 4–6 months post‐surgery, in conjunction with intensive physical therapy, by three SCI survivors who could walk with the assistance of a walker (Rowald et al., 2022).

To support the development of novel neural prostheses and rehabilitative therapies, there is a need for a better understanding of the anatomical and physiological basis of weight bearing. Pioneering electromyography work has identified key postural muscles during standing (Basmajian & De Luca, 1985); studies repeatedly highlighted the role of the posterior leg muscles (Joseph et al., 1955;Joseph & Nightingale, 1952; Morin & Portnoy, 1956) and some noted activation in muscles of the posterior thigh, lower back and abdomen (Asmussen, 1959; Morin & Portnoy, 1956). Common limitations of these past investigations have been small sample size, large inter‐individual variation and a limited statistical analysis (Joseph & Nightingale, 1952; Morin & Portnoy, 1956). There has been little work in this field since this point, in part because there has been limited clinical need for further knowledge. The limitations of these past studies, in tandem with modern, more sensitive equipment and an increased clinical demand, necessitate further systematic investigation into the basic biomechanics of independent standing.

This pilot study considers the fundamental role of weight bearing in independent standing. We tested healthy individuals, recording multiple muscles at different body angles, to investigate efficient standing and to determine responses at different gravitational loads. Recording muscle activation at different body angles, with 15 deg intervals, probes the system and allows us to measure subtle changes. This reduces the need for extremely high precision, invasive technology and large participant numbers. By recording surface muscle activity, this study aims to provide new, granular evidence of the muscle and spinal activation required at different gravitational loads.

## 2 MATERIALS AND METHODS

### 2.1 Ethics statement

Participants recruited for this study provided written informed consent to all protocols and methods outlined prior to starting the experiment. The experiment was in accordance with the Declaration of Helsinki and was approved and conformed to the standards outlined by the King's College London ethics committee (MRA‐19/20‐14,388). Participants over 18 years of age and with no history of neurological or musculoskeletal injury or surgery within the previous year were recruited.

### 2.2 Participants and procedure

Muscle activity was recorded in 18 healthy participants, 11 males and seven females. The mean age, height and weight of the participants were 23.6 ± 8.4 years (mean ± standard deviation), 1.722 ± 0.122 m and 70.0 ± 15.8 kg respectively. Participants attended one laboratory visit in which muscle activation was recorded using surface electromyography (EMG). The eight available EMG electrodes (Avanti, Delsys Inc.) were placed on 19 muscles unilaterally on the non‐dominant side of each participant's body, over three sessions as summarised in Table 1. The position of EMG electrodes was optimised by investigating cadaveric samples before the beginning of the study; this established accessibility of the muscles, their size to accommodate electrodes and the orientation of the muscle fibres.

**TABLE 1:** Muscles tested in each of the sessions (session 1, 2 and 3). Muscles were tested unilaterally on the non‐dominant side of the participant's body

	Session 1	Session 2	Session 3
Muscles	Soleus	Soleus	Soleus
	Gastrocnemius medialis	Rectus femoris	Gracilis
	Gastrocnemius lateralis	Sartorius	Rectus abdominis
	Tibialis anterior	Extensor digitorum longus	External oblique
	Adductor longus	Semimembranosus	Erector spinae
	Peroneus longus	Semitendinosus	Latissimus dorsi
	Vastus medialis	Biceps femoris (long head)	
	Vastus lateralis		

*Note*: Selection of muscles to be studied in each session was made to minimise electrode crosstalk. Soleus was included in all sessions as a control.

When testing healthy participants, the position of each muscle was identified, the skin was prepared (shaved and abraded using a razor, and then cleaned with an alcohol swab) and the electrode was placed using custom adhesives. Successful electrode positioning was confirmed by asking participants to carry out muscle‐specific movements and inspecting the muscle activity in real time (Vicon). Our work on cadaveric material and real‐time verification after electrode placement was necessary to assure robust electrodes' placement, irrespective of anatomical inter‐personal differences. Participants then lay barefoot on a motorised plinth (SEERS Medical Ltd.) and were asked to look straight ahead. Participants kept their arms by their sides, had their feet hip width apart and adopted a relaxed position without unnecessary gross movement.

**FIGURE 1:**
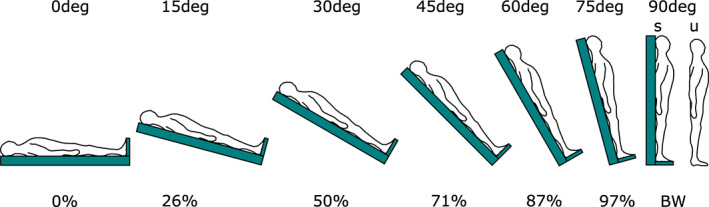
Experimental protocol, indicating body orientation in degrees and approximate percentage of body weight (BW). Participants were supine on a motorised plinth which was moved from 0 deg to 90 deg body orientation, at 15 deg increments. The load experienced at the different body angles was 0% of body weight (BW) at 0 deg, ~26% BW at 15 deg, 50% BW at 30 deg, ~71% BW at 45 deg, ~87% BW at 60 deg, ~97% BW at 75 deg and 100% BW at supported (s90deg) and unsupported 90 deg (u90deg).

Muscle activity was recorded with the participant supine (0 deg) and then the motorised plinth was moved to the subsequent body orientation, measured by the inclinometer on the plinth. Eight body orientations, at 15 deg increments, were tested (Figure 1). Standing activity was measured both on the plinth (supported, hereafter **s**90deg) and during quiet standing on the floor (unsupported, hereafter **u**90deg). The different body angles induced different vertical loadings at the feet, which were calculated by the participant's weight and the sine of the body angle. The load experienced would be 0% of body weight (BW) at 0 deg, ~26% BW at 15 deg, 50% BW at 30 deg, ~71% BW at 45 deg, ~87% BW at 60 deg, ~97% BW at 75 deg and 100% BW at s90deg and u90deg.

For each test angle, muscle activity was recorded for 30 s at 2040 Hz sampling frequency. Non‐randomised, incremental body angles were preferred to minimise participants' discomfort when being passively moved between positions on the plinth. A 10‐s resting period after rotating the plinth to the new angle allowed for participants to adjust to the new position before measurements were taken. When one session (eight body angles) was concluded, the plinth was returned to 0 deg to start a new session. The procedure, including a 1–2 min break and skin preparation and electrodes placement verification, was repeated with the three‐electrode configurations in the three sessions (Table 1). The time between sessions was approximately 5–10 min.

### 2.3 Data analysis

The EMG data were exported as text files and analysed using custom‐written Matlab scripts (MathWorks). The raw signal was rectified and filtered through a high‐pass fourth‐order Butterworth filter with a cut‐off frequency of 10 Hz, a fourth‐order Butterworth low‐pass filter with a cut‐off frequency of 500 Hz and a notch filter at 50 Hz. The mean muscle activation was calculated for the data between 5 and 25 s of the 30 s recording. This removed any filtering artefacts and any muscle activation due to involuntary movement (e.g., adjusting body position for comfort or fatigue) from participants at either end of the recording. Data observation indicated that some participants showed lower activity during unsupported standing compared to baseline when supine on the plinth. This was consistent with participants failing to relax on the plinth and introducing a bias in the group results. To identify clear outliers, we used a stringent criterion assuming that if a participant showed lower muscle activation during unsupported standing compared to baseline in more than 12 muscles (half of all the 19 muscles studied +1, considering that soleus was recorded in all the sessions), that participant should be excluded from further analysis. Using this criterion, two participants, participant 5 and 14, were excluded from the analysis.

The mean EMG data from each muscle was used to compute the spinal activation profiles (Ivanenko et al., 2006) using the formula:

$$\text{Spinal segment activation}=\frac{\sum k*\text{EMGi}}{n}$$

Where k was 0.5 or 1 according to accepted values published by Kendall (Kendall et al., 1993). Considering the muscles included in this study, the following spinal sections are reported: cervical C6–C8, thoracic T5 to T6 and T7–T12, lumbar (L1, L2–L3, L4 and L5) and sacral segments (S1, S2). The EMG for soleus was recorded in all three trials, acting as a control to identify any salient changes due to recording different muscles in different sessions. The three recordings from soleus were averaged when calculating the spinal activation. For each spinal level, the exact calculations with the muscles included in this study are reported below, where *K* = 1, *k* = 0.5.

$$C6\_\text{C8}=\left(\text{Latissimus}\_{\text{Dorsi}}^{*}\;K\right)/1$$

$$\begin{array}{c} T5\_6=\left(\text{Erector}\_{\text{Spinae}}^{*}\;K+\text{External}\_{\text{Oblique}}^{*}\;k\right.\\ \left.\;+\text{Rectus}\_{\text{Abdominis}}^{*}\;K\right)/3 \end{array}$$

$$\begin{array}{c} T7\_12=\left(\text{Erector}\_{\text{Spinae}}^{*}\;K+\text{External}\_{\text{Oblique}}^{*}\;K\right.\\ \left.\;+\text{Rectus}\_{\text{Abdominis}}^{*}\;K\right)/3 \end{array}$$

$$\text{L1}=\left(\text{Erector}\_{\text{Spinae}}^{*}\;K\right)/1$$

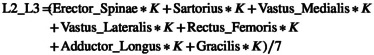




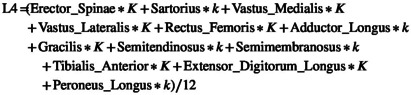




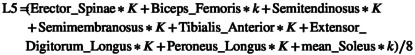

$$\begin{array}{c} \begin{array}{cc} S1=\; & (\text{Erector}\_\text{Spinae}*K+\text{Biceps}\_\text{Femoris}*K+\text{Semitendinosus}*K\\ & +\text{Semimembranosus}*K+\text{Tibialis}\_\text{Anterior}*k+\text{Extensor}\_\\ & \text{Digitorum}\_\text{Longus}*K+\text{Peroneus}\_\text{Longus}*K+\text{Gastrocnemius}\_\\ & \text{Medialis}*K+\text{Gastrocnemius}\_\text{Lateralis}*K+\text{mean}\_\text{Soleus}*K)/10 \end{array} \end{array}$$

$$\begin{array}{c} \begin{array}{cc} S2=\; & (\text{Erector}\_\text{Spinae}*K+\text{Biceps}\_\text{Femoris}*K+\text{Semitendinosus}*K+\\ & \text{Semimembranosus}*k+\text{Gastrocnemius}\_\text{Medialis}*K+\\ & \text{Gastrocnemius}\_\text{Lateralis}*K+\text{meanSoleus}*K)/7 \end{array} \end{array}$$

### 2.4 Statistical analysis

The EMG and spinal activation levels are reported relative to those calculated when the body was at 0 deg for each participant, by subtracting the baseline activity measured at 0 deg. This baseline individual normalisation choice is justified to allow averages calculation and to remove individual participant bias due to skin preparation, anatomical differences (type and depth of non‐muscular tissue) and resting tonic levels. The relative EMG and spinal activation levels from each participant were included in the statistical analysis using Matlab Statistics and Machine Learning Toolbox (MathWorks). A non‐parametric Kruskal–Wallis test was used. For the muscle and spinal activation measures, the factors studied were the different body angles (eight levels). Tukey–Kramer post hoc test compared the results at the different body angles and spinal levels relative to the 0 deg (baseline) and 15 deg body angle. The 15deg body angle was included, as it was the not‐null, but lowest body loading, to confirm and check the activation recorded when laying supine at 0 deg. No other pairwise comparisons are reported. The group relative EMG activity is presented as mean ± standard error of the mean from all participants. Spinal activation is presented as mean and median of the group. Statistical significance was set at *p* < 0.05.

## 3 RESULTS

### 3.1 Group muscle activation

**FIGURE 2:**
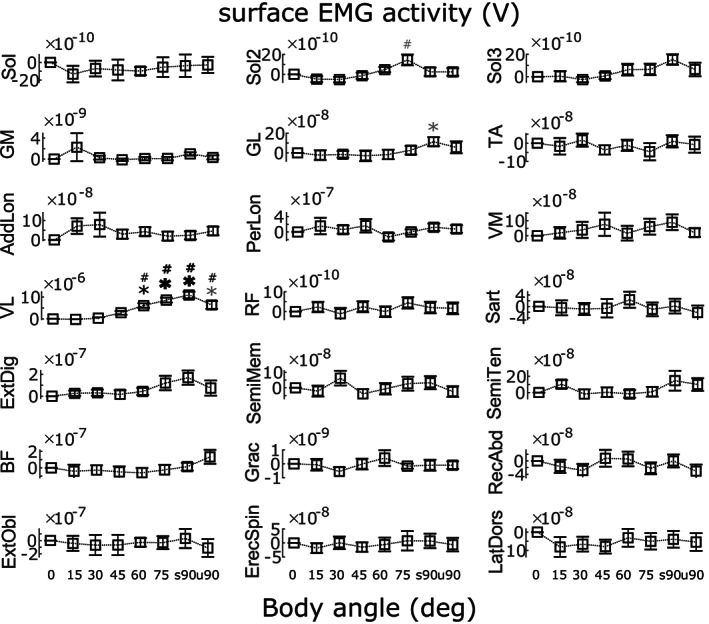
Group mean ± SEM EMG (V) of all participants' muscles recorded relative to 0 deg (calculated for each participant independently, *n* = 16). The results are shown for all the body angles tested (0‐u90deg). Soleus (Sol) is reported three times because it was recorded as control in all the three sessions. Muscles included: soleus (Sol), gastrocnemius medialis (GM) and lateralis (GL), tibialis anterior (TA), adductor longus (AddLon), peroneus longus (PerLon), vastus medialis (VM) and lateralis (VL), rectus femoris (RF), sartorius (Sart), extensor digitorum longus (ExtDig), semimembranosus (SemiMem), semitendinosus (SemiTen), biceps femoris (BF), gracilis (Grac), rectus abdominus (RecAbd), external oblique (ExtObl), erector spinae (ErecSpin), latissimus dorsi (LatDors). For comparisons relative to 0 deg: * (grey) represents *p* < 0.05, * (black) represents *p* < 0.01 and * (bold) represents *p* < 0.001. For comparisons relative to 15 deg, # (grey) represents *p* < 0.05, # (black) represents *p* < 0.01 and **#** (bold) represents *p* < 0.001. All the specific results are reported below. Vastus lateralis was more active at 60 deg, 75 deg, s90deg and u90deg compared to 0 deg (p_0_15deg_ = 0.9926; p_0_30deg_ = 1; p_0_45deg_ = 0.4826; p_0_60deg_ = 0.0043; p_0_75deg_ <0.0001; p_0_s90deg_ <0.0001; p_0_u90deg_ = 0.0102) and 15 deg (p_15_30deg_ = 0.9892; p_15_45deg_ = 0.0925; p_15_60deg_ = 0.0001; p_15_75deg_ <0.0001; p_15_s90deg_ <0.0001; p_15_u90deg_ = 0.0004). Gastrocnemius lateralis was more active at s90deg compared to 0 deg (p_0_15deg_ = 0.9999; p_0_30deg_ = 1; p_0_45deg_ = 0.9949; p_0_60deg_ = 0.9969; p_0_75deg_ = 0.3154; p_0_s90deg_ = 0.0469; p_0_u90deg_ = 0.7372), but not compared to 15 deg (p_15_30deg_ = 1; p_15_45deg_ = 1; p_15_60deg_ = 1; p_15_75deg_ = 0.5784; p_15_s90deg_ = 0.1376; p_15_u90deg_ = 0.9259). For soleus recorded in session 2, no difference was found in activation when compared to 0 deg (p_0_15deg_ = 0.9969; p_0_30deg_ = 0.9832; p_0_45deg_ = 1; p_0_60deg_ = 0.9743; p_0_75deg_ = 0.1739; p_0_s90deg_ = 0.9933; p_0_u90deg_ = 0.9503), but activation was higher at 75 deg compared to 15 deg (p_15_30deg_ = 1; p_15_45deg_ = 0.9995; p_15_60deg_ = 0.6823; p_15_75deg_ = 0.0248; p_15_s90deg_ = 0.8064; p_15_u90deg_ = 0.5949). While no differences were found for soleus recorded in session 3 compared to 0 deg (p_0_15deg_ = 1; p_0_30deg_ = 0.9881; p_0_45deg_ = 0.9998; p_0_60deg_ = 0.9811; p_0_75deg_ = 0.7826; p_0_s90deg_ = 0.3463; p_0_u90deg_ = 0.9990), or compared to 15 deg (p_15_30deg_ = 0.9990; p_15_45deg_ = 1; p_15_60deg_ = 0.9215; p_15_75deg_ = 0.5982; p_15_s90deg_ = 0.1974; p_15_u90deg_ = 0.9884). Gastrocnemius medialis showed a trend in significance, but no difference was found in activation when compared to 0 deg (p_0_15deg_ = 0.9357; p_0_30deg_ = 1; p_0_45deg_ = 0.9070; p_0_60deg_ = 0.9943; p_0_75deg_ = 0.9743; p_0_s90deg_ = 0.6823; p_0_u90deg_ = 1), but s90deg showed a tendency for higher activation when compared to 15 deg (p_15_30deg_ = 0.9433; p_15_45deg_ = 1; p_15_60deg_ = 0.9999; p_15_75deg_ = 1; p_15_s90deg_ = 0.0734; p_15_u90deg_ = 0.8947).

The muscle activation relative means from all participants included (*n* = 16) are reported in Figure 2. A significant difference in activation level according to angle was found for several muscles. As reported in Table 2, the muscle activation was significantly different according to body angle for vastus lateralis, gastrocnemius lateralis and soleus in session 2 and 3. Gastrocnemius medialis showed results close to significance. No significant difference was found for the other muscles. For the significant muscles, Tukey–Kramer post hoc analysis relative to 0 deg and 15 deg body angle was run. These results are reported immediately below and in the figures.

**TABLE 2:** Results of the Kruskal–Wallis test for all the muscles, including chi‐square and overall *p*‐values for muscle activation at the different body angles

	*χ* ^2^(7)	Factor: Body angle
		*p*
Latissimus Dorsi	5.72	0.5729
Erector Spinae	1.68	0.9754
External Oblique	5.55	0.5931
Rectus Abdominis	5.97	0.5434
Gracilis	3.67	0.8172
Sartorius	4.02	0.7775
Adductor Longus	5.64	0.5830
Rectus Femoris	3.07	0.8784
Vastus Medialis	4.09	0.7689
Vastus Lateralis	**81.62**	**<0.001**
Biceps Femoris	4.66	0.7008
Semitendinosus	4.96	0.6651
Semimembranosus	8.90	0.2602
Soleus session 1	6.95	0.4338
Soleus session 2	**18.30**	**0.0107**
Soleus session 3	**15.80**	**0.0270**
Peroneus Longus	6.35	0.4992
Gastrocnemius Lateralis	**15.89**	**0.0262**
Gastrocnemius Medialis	**13.68**	**0.0572**
Extensor Digitorum	5.42	0.6086
Tibialis Anterior	4.85	0.6778

*Note*: Results of the post hoc comparisons are reported in the text. The muscles highlighted in bold showed a *p*‐value < 0.05.

Two muscles showed increased activation compared to 0 deg body angle. Vastus lateralis was more active at 60 deg, 75 deg, s90deg and u90deg (p_0_60deg_ = 0.0043; p_0_75deg_ <0.0001; p_0_s90deg_ <0.0001; p_0_u90deg_ = 0.0102). Gastrocnemius lateralis was more active at s90deg compared to 0 deg (p_0_s90deg_ = 0.0469). For soleus recorded in session 2 and 3, and gastrocnemius medialis, no difference was found in activation when compared to 0 deg.

Similar results were seen for these muscles relative to 15 deg. Vastus lateralis' activation was higher at 60 deg, 75 deg, s90deg and u90deg (p_15_60deg_ = 0.0001; p_15_75deg_ <0.0001; p_15_s90deg_ <0.0001; p_15_u90deg_ = 0.0004). For soleus recorded in session 2, activation was higher at 75 deg compared to 15 deg (p_15_75deg_ = 0.0248), but no difference was found for soleus recorded in session 3. No difference was found for gastrocnemius lateralis, while a gastrocnemius medialis at s90deg showed a tendency for higher activation when compared to 15 deg (p_15_s90deg_ = 0.0734).

### 3.2 Group spinal activation

**FIGURE 3:**
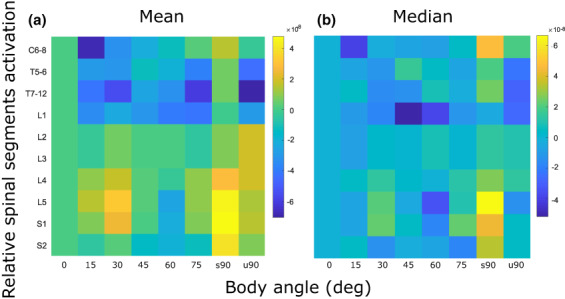
(**a**) Mean and (**b**) median spinal activation map from the individual participants' spinal activation (*n* = 16), relative to the activation at 0 deg. The spinal segments considered are shown on the vertical axis and the results are shown for all the body angles tested (0‐u90deg) on the horizontal axis. An angle effect was measured for the lumbar level L5 (*χ* ^2^(7) = 15.11, *p* = 0.0346) and the sacral S1 levels (*χ* ^2^(7) = 14.37, *p* = 0.0450). For L5, post hoc analysis showed no significant differences relative to 0 deg (p_0_15deg_ = 1; p_0_30deg_ = 0.9330; p_0_45deg_ = 1; p_0_60deg_ = 0.9480; p_0_75deg_ = 0.9990; p_0_s90deg_ = 0.2706; p_0_u90deg_ = 1) or 15 deg (p_15_30deg_ = 1; p_15_45deg_ = 0.8190; p_15_60deg_ = 0.9895; p_15_75deg_ = 0.9888; p_15_s90deg_ = 0.1473; p_15_u90deg_ = 0.9994). For S1, post hoc analysis showed no significant differences relative to 0 deg (p_0_15deg_ = 1; p_0_30deg_ = 0.9816; p_0_45deg_ = 1; p_0_60deg_ = 0.9722; p_0_75deg_ = 0.9986; p_0_s90deg_ = 0.2509; p_0_u90deg_ = 0.9997) or 15 deg (p_15_30deg_ = 0.8966; p_15_45deg_ = 1; p_15_60deg_ = 0.9981; p_15_75deg_ = 0.9770; p_15_s90deg_ = 0.1100; p_15_u90deg_ = 0.9899).

The mean and median spinal activation maps relative to the activation measured at 0 deg are reported in Figure 3. Median spinal activation is included to show the potential effect of individual variation in the sample. Clearly, the representation of these results affects the spinal map. The statistical analysis was run on the group data obtained from the single participants' mean activation, and results for spinal segment L2 and L3 were merged, as the muscles and formula used for these segments was the same (nine spinal segments tested).

An angle effect was measured for the lumbar level L5 (*χ* ^2^(7) = 15.11, *p* = 0.0346) and for the sacral level S1 (*χ* ^2^(7) = 14.37, *p* = 0.0450). For both segments, post hoc analysis showed that activations at 0 deg or 15 deg were not different from the other angles. No angle effect was measured for the cervical, thoracic or the other lumbar and sacral segments (C6‐C8: *χ* ^2^(7) = 5.07, *p* = 0.6510; T5–T6: *χ* ^2^(7) = 6.49, *p* = 0.4836; T7–T12: *χ* ^2^(7) = 8.48, *p* = 0.2923; L1: *χ* ^2^(7) = 2.41, *p* = 0.9337; L2–L3: *χ* ^2^(7) = 3.32, *p* = 0.8544; L4: *χ* ^2^(7) = 12.19, *p* = 0.0945; S2: *χ* ^2^(7) = 7.56, *p* = 0.3734).

## 4 DISCUSSION

In this study, the EMG activation of 19 superficial muscles was recorded at different body angles, and maps of spinal activation for different body loading and quiet standing were produced. We found that the muscles that were activated for weight bearing were recruited when the body angle was equal or greater than 75 deg (~97% bodyweight), and vastus lateralis became active at 60 deg (~87% bodyweight). Spinal activity maps showed that the highest modulation of activity was in the L5 and S1 regions.

These results are considered preliminary because testing was halted due to the Covid‐19 pandemic. The relatively small number of participants for a study recording tonic EMG activity may affect the results due to inter‐individual variation. However, non‐parametric analysis and the calculation of median spinal maps from 16 participants suggest two key findings: (i) independent standing can be achieved with increased activation of a limited number of muscles, and (ii) lumbar and sacral segments are more likely to be involved in maintaining weight‐bearing standing than other spinal segments. Here, we discuss these findings and their implications.

### 4.1 Muscle activation in response to increased body load

Independent bipedal standing is a complex task. In this study, we focused on the tonic muscle activation required to maintain balance with an increased gravitational load from supine to standing. We found that tonic activity increased when loading was equal to 97% and 87% total body weight, corresponding to 75 deg and 60 deg body angles respectively. During gait, body loading on either limb ranges from ~60% to ~120% of body weight, depending on the phases of the gait cycle (Bonnefoy‐Mazure & Armand, 2015; MacConaill & Basmajian, 1977). Our results suggest that increasing the activation in vastus lateralis, gastrocnemius lateralis and medialis, and soleus could sustain the loading of standing, and possibly walking. These results do not mean that the other muscles recorded were inactive, but simply that their activation level was not statistically different from the level recorded when supine.

The role of the gastrocnemii and soleus muscles in standing has been investigated extensively (Basmajian & De Luca, 1985; Di Giulio et al., 2009; Joseph et al., 1955; Loram et al., 2007; Loram & Lakie, 2002; Morin & Portnoy, 1956). In our experiments, soleus was used as a control muscle across the three sessions. In sessions 2 and 3, we found an overall difference. However, the post hoc pairwise comparisons were not entirely consistent. The gastrocnemii are ankle plantarflexors and knee flexors, and soleus is the most established postural muscle. However, our inconsistent results could be explained by different, complementary hypotheses. It is possible that the resting tonic activation in soleus, even when supine, was equivalent to the activation level during standing; therefore, no increase was needed in standing positions. It is also possible that participants were unable to completely relax soleus and/or that their ankle/body position on the plinth caused measurable activation, resulting in soleus' recorded activity even when supine. This hypothesis may explain why a difference in soleus' activation was found in session 2 and 3, since participants may have become familiar with the experimental set‐up and were more able to relax. This inconsistency in our results led us to conclude that there may be a minimal difference in soleus activation level as body loading increased, and future experiments should dedicate more time to protocol familiarisation with the participants. Recording at least one control muscle in all the sessions is a useful method to interpret the results.

The recorded increased activation of vastus lateralis, a lateral hip stabiliser and knee extensor, has an antagonist effect to gastrocnemii’ activation. The combined activity of these muscles may be consistent with maintaining the knee's efficient, close‐packed position during standing (MacConaill & Basmajian, 1977). It is also likely that vastus lateralis' activation contributes to hip stability during standing. Hip extension is an unresolved issue with epidural spinal cord stimulation of SCI survivors (Rejc et al., 2015), and here, we provide anatomical evidence suggesting that quadriceps muscles' activation may be needed for successful weight bearing. Contrary to our expectations, we did not find increased activation in the muscles of the posterior compartment of the thigh. However, our analysis relative to baseline only indicates that a difference from supine position was not measured. It is possible that tonic activation of these muscles at baseline was already close to the one needed for standing. It is also possible that our study was underpowered to measure these particular changes effectively. These constraints of the study should be considered in light of other limitations. Choosing to focus on the lower limb muscles reflects the overarching aim of this investigation, which was to provide anatomical and physiological evidence of the tonic activation of superficial muscles, with the long‐term objective of informing spinal stimulation protocols for SCI survivors. Our work focused on the effect of lower spinal lesions, where the need for independent standing and walking is more prominent, and the upper body function is unaffected. We believe that individuals with higher level lesions may be more concerned with restoring functions other than gait to improve their quality of life.

Furthermore, we decided to only record superficial muscles to avoid interfering with the functional anatomy using multiple needle recordings. Additionally, some muscles were excluded because they would be uncomfortable for the participants, especially when supine, such as the gluteus maximus. Using a motorised plinth to rotate the participants and increase gravitational loading rather than a harness‐based unloading system was convenient in our laboratory. This approach was preferred to minimise participants' discomfort and to replicate some of the scenarios tested in the rehab clinic with different patient groups (e.g., stroke and SCI survivors). We expect the physiological responses to different gravitational loads to be similar, irrespective of the method used to apply the different (un)loading. However, we could not test this assumption or determine whether friction between the participant and the plinth influenced the effective loading experienced by our participants. More work with harness‐based unloading may provide additional insight in the mechanisms involved in weight bearing. Finally, despite our efforts of excluding participants that consistently showed lower activation in muscles when standing unsupported compared with baseline and repeating trials when participants moved or reported that they were not fully relaxed when supine, we cannot exclude that other participants were not fully relaxed, and this may have affected the results presented here.

### 4.2 Lumbar and sacral spinal segments were activated with increased body load

The results from the spinal map calculations showed that the L5 and S1 segments' activation was modulated in response to changes in body loading more than the other segments. The specific activation of these segments is interesting because central pattern generators for locomotion, which are thought to generate the rhythmic stepping movements of gait, are thought to be located in the lumbar region, specifically in the high lumbar region around the second lumbar spinal segment (Cina & Hochman, 2000; Dimitrijevic et al., 1998; Kjaerulff et al., 1994; Nishimaru et al., 2000). Our study focused on understanding the spinal activation for independent standing to provide evidence to sustain independent locomotion. Here, we show that the lower lumbar and higher sacral segments are active with changes in body loading. These results may relate to previous studies on locomotion development, since Ivanenko and his group showed a shift in spinal activation from the higher spinal segments in neonates (L2–L4) towards lower spinal segment (L4–S2) in adults (Ivanenko et al., 2013). There are two main considerations to address when comparing the results from Ivanenko and colleagues with the current ones. Firstly, the neonates in Ivanenko's study had to be supported by an operator, therefore, the activation measured at higher lumbar segments may be related to the stepping actions, while the distal shift in activation seen with maturation may be explained by the activation needed for weight bearing. Secondly, in adults, the higher spinal segments (L4–L5) were activated in the first part of stance at touch down, and therefore during double support, while the lower segments (S1–S2) were activated in middle‐late stance, during single support. The activation of the lower segments in single support is consistent with our results indicating that increased lower spinal activation is necessary to sustain increased weight bearing.

Our results provide new anatomical and physiological evidence to support the research into restoring independent standing, locomotion, weight bearing and load shift. For gait restoration, epidural stimulation is thought to work in tandem with central pattern generators in the spinal cord (Minassian et al., 2016), but fully independent standing and locomotion appear to be elusive. Epidural stimulation of L5 and S1 elicited stepping movements with the need for body support in patients with SCI (Harkema et al., 2011), while stimulation of segments T12–S2 achieved locomotion with a walker, after 4–6 months of intense rehabilitation (Rowald et al., 2022). Our results suggest that tonic activation of L5 and S1 is likely to be required for weight bearing. Adjusting the stimulation pattern based on our evidence may improve the outcomes of spinal stimulation protocols and target the stimulation efficiently for different body loading levels and during weight shifting, which are typical of activities of daily living such as sit‐to‐stand, stair climbing or obstacle negotiation.

To conclude, we found an increased activation of vastus lateralis, gastrocnemius lateralis and soleus in standing compared to the supine position. The calculated spinal activation showed increased activity during standing in the lower lumbar and higher sacral segments. This study shows that 87% body weight is a critical loading for increased muscle activation, compared with the fully supported supine position. Our findings could inform rehabilitation protocols on muscular activation according to weight‐bearing levels, as often these approaches use body support. Here, we provide granular evidence for muscles activated at different loading levels. We also identified critical body loading levels for muscle recruitment and the data are also relevant to different activities of daily life when transitioning between positions, for example from sitting to standing, where the loading varies throughout the movement. Our work suggests that the lower lumbar and higher sacral segments are activated for weight bearing. We speculate that to achieve independent gait, the higher lumbar segments are needed for the rhythmic stepping movements and the lower lumbar and sacral segments are needed for independent body support.

## AUTHOR CONTRIBUTIONS

**BC**: data collection and analysis, manuscript writing and editing; **JAH**: data collection and analysis, manuscript writing and editing; **IDG:** conceptualisation of the experiment, experimental set‐up, data analysis, manuscript writing, review and editing.

## CONFLICT Of INTEREST

All authors declare that they have no conflicts of interest.

## Data Availability

The raw data collected for this study are available upon request, by contacting the corresponding author.
